# Is there a need for neoadjuvant short-course radiotherapy in T3 rectal cancer with positive lymph node involvement? A single-center retrospective cohort study

**DOI:** 10.1186/s12957-019-1670-0

**Published:** 2019-08-08

**Authors:** Minna Räsänen, Laura Renkonen-Sinisalo, Harri Mustonen, Anna Lepistö

**Affiliations:** 1 Colorectal Surgery, Abdominal Centre, Helsinki University Hospital, Haartmaninkatu 4, PL340, 00029 HUS, Helsinki, Finland; 2 Department of Medicine, University of Helsinki, Helsinki, Finland; 3 Research Programs Unit, Genome-Scale Biology, University of Helsinki, Helsinki, Finland

**Keywords:** Rectal cancer, pT3N1-2 rectal cancer, Short-course radiotherapy, Local recurrence

## Abstract

**Background:**

Neoadjuvant short-course radiotherapy is used to reduce local recurrences in stage III rectal cancer. Radiotherapy is not harmless, and meticulous total mesorectal excision surgery alone has been reported to result in low local recurrence rate in favorable stage III tumors. The aim was to evaluate the effect of short-course (5 × 5 Gy) radiotherapy on the local recurrence risk in patients with pT3N1-2 rectal cancer.

**Materials and methods:**

This was a retrospective study with 151 consecutive pT3N1-2M0 rectal cancer patients operated on at Helsinki University Hospital, Helsinki, Finland, during January 2005 to June 2014. Short-course radiotherapy was given to 94 patients, and 57 patients were operated on without neoadjuvant radiotherapy. The main outcome measurement was the effect of radiotherapy on local recurrence. Also, the risk factors for local recurrence were analyzed.

**Results:**

Local recurrence occurred in a total 17 of 151 (11.3%) patients, 8 of 57 (14.0%) in surgery only group compared with 9 of 94 (9.6%) in radiotherapy plus surgery group (*p* = 0.44). In univariate Cox regression analysis, the risk factors for local recurrence were tumor location under 6 cm from the anal verge (*p* = 0.01), involved lateral margin (*p* < 0.001), tumor perforation (*p* < 0.001), and mucinous histology (*p* = 0.006). In multivariate analysis, risk factors were tumor location under 6 cm from anal verge (*p* = 0.03) and involved lateral margin (*p* = 0.002).

**Conclusion:**

Neoadjuvant short-course radiotherapy did not affect the local recurrence risk of pT3N1-2M0 rectal cancer. Further studies with larger patient number are needed to evaluate the role of short-course radiotherapy in different T3 subgroups (3a–c) as well as in N1 and N2 cancers in separate.

## Introduction

Local recurrence was a major problem in rectal cancer before the introduction of the total mesorectal excision (TME). Swedish rectal cancer trial showed that neoadjuvant short-course radiotherapy (RT) reduced the local recurrence risk and improved survival in patients operated on before the adoption of the TME technique [1, 2]. Dutch trial found that short-course radiotherapy reduced the risk of local recurrence when combined with TME surgery, however, without any beneficial effect on survival [3–5]. It was reported that there was 10.6% local recurrence risk in stage III disease after short-course RT combined with TME and 20.6% local recurrence risk in those treated with TME alone [4]. On the other hand, a local recurrence rate of 4% with meticulous TME surgery alone has been reported and no difference in local recurrence rate between stage II and III disease was noted in that series [6–8]. Efforts have also been made to recognize the so-called “good” prognosis T3 cancers and separate them from “poor” prognosis T3 cancers [8, 9]. However, we do not know if the patients with ugly T3 cancer benefit from short-course RT.

Short-course RT can cause harm. It increases the leakage risk of colorectal anastomosis, and it can cause bowel, urinary, and sexual dysfunction [10–14]. It is also suspected to have a higher incidence of secondary malignancies after radiotherapy [15].

At the present, there are remarkable differences in preoperative treatment strategies of rectal cancer in European countries [16]; Sweden and the Netherlands use neoadjuvant short-course RT most often, but in Norway and Denmark, short-course RT is little used as a treatment option. Despite this, there are no remarkable differences between the oncological treatment results of these countries.

The treatment strategy of rectal cancer in our unit is very similar to that of Sweden; most patients with T3 tumors and a suspicion of positive lymph node involvement receive short-course neoadjuvant RT. However, diagnosis of positive lymph node involvement in rectal MRI is difficult, as shown by high-quality units [8], and some patients are not suitable for RT for other reasons; therefore, there are still substantial numbers of patients with pT3N1-2 rectal cancer whom have not undergone short-course RT preoperatively.

The first aim of this study was to evaluate the effect of short-course RT on local recurrence risk in patients that have pT3N1-2 rectal cancer in pathological specimen. The second aim was to compare risk factors between patients that had short-course neoadjuvant RT before TME surgery and those that had TME surgery alone.

## Materials and methods

### Patient characteristics

A total of 151 patients, having had pT3N1-2M0 rectal cancer and TME, operated on in Helsinki University Hospital over the beginning of 2005–June 2014 period were included in retrospective analysis. In all, 952 patients were operated on for rectal cancer in our unit during this same time period. Short-course neoadjuvant radiotherapy (5 × 5 Gy) was given to 94 patients, and they were operated on within 5 days after RT (the RT plus surgery group). Fifty-seven patients were operated on without neoadjuvant therapy (the surgery only group). Patients underwent whole-body computer tomography (CT) and magnetic resonance imaging (MRI) of the rectum for the preoperative staging of disease. Histology of the tumor was subsequently verified by preoperative endoscopy biopsies. The necessity of neoadjuvant RT was determined in a multidisciplinary team meeting that comprised colorectal surgeon, an oncologist, a radiologist, and a pathologist. The patients, who were evaluated to have rT2-T3N0 tumor on the basis of preoperative MRI, did not receive RT. Some patients with rT3N1-2 tumor were not able to be treated with neoadjuvant RT because of previous pelvic RT for other reasons. The CRM was not threatened based on MRI in the study group. The data were collected from patient records after at least 2 years of follow-up.

The median follow-up time for the whole patient group was 4.3 (range 0.01–11.3) years. The follow-up time was defined as time in years from the day of surgery until the last contact day with the health care system or death. The cause of death was verified from official death certificates. One patient died during the 30-day postoperative period due to pulmonary embolism. This patient was excluded only from the risk factor analysis for local recurrence. No patient was lost to follow-up. The local ethics committee approved the study protocol. Patient and disease characteristics are shown in Tables 1 and 2.

**Table 1:** Patient characteristics

Characteristics	Surgery only, *N* = 57 (%)	Radiotherapy plus surgery, *N* = 94 (%)	*p*
Follow-up time			0.62
Median (years)	4.0	4.4	
Range (years)	0.2–11.2	0.01–11.3	
Age			0.01
Median (years)	71	66	
Range (years)	32–88	37–86	
Gender			0.87
Male	28 (49)	44 (47)	
Female	29 (51)	50 (53)	
BMI			1.0
≤ 25 kg/m^2^	23 (40)	44 (47)	
> 25 kg/m^2^	19 (33)	38 (40)	
Missing^a^	15 (27)	12 (13)	
Tumor distance from anal verge			0.17
≤ 6 cm	17 (30)	39 (42)	
> 6 cm	40 (70)	55 (58)	
Type of surgery			0.10
Anterior resection (AR)	40 (70)	78 (83)	
Abdominoperineal excision (APE)	11 (19)	13 (14)	
Hartmann’s procedure	6 (11)	3 (3)	
Macroscopically curative surgery	56 (98)	93 (99)	1.0
Postoperative chemotherapy	35 (61)	82 (87)	< 0.001
Death	24 (42)	27 (29)	0.11
Cancer-related death	17 (30)	20 (21)	0.25

^a^Data not available, not included in *p* level count

**Table 2:** Disease characteristics

Characteristics	Surgery only, *N* = 57 (%)	Radiotherapy plus surgery, *N* = 94 (%)	*p*
Histology			0.76
Adenocarcinoma	48 (84)	82 (87)	
Mucinous carcinoma	9 (16)	11 (12)	
Neuroendocrine carcinoma	0	1 (1)	
Grade			0.87
1	4 (7)	5 (5)	
2	41 (72)	74 (79)	
3	10 (18)	13 (14)	
4	0 (0)	2 (2)	
Missing^a^	2 (3)	0	
pN			0.62
1	33 (58)	50 (53)	
2	24 (42)	44 (47)	
Lateral margin R1 (< 1 mm)	6 (11)	6 (6)	0.37
Tumor perforation	5 (9)	2 (2)	0.10
Invasion			0.04
Vascular/neural/lymphatic	15 (26)	41 (44)	

^a^Data not available, not included in the significance test

The rectal cancer was operated on using the TME technique; the operation in the upper rectum was by partial mesorectal excision (PME). A colonic J-pouch was routinely constructed in operations for middle and low rectal tumors. The covering stoma was performed in 121 patients. The macroscopic result was considered compromised if either spontaneous or iatrogenic tumor perforation occurred or if the surgeon thought that the resection margin might be involved. Tumor location 6 cm or under from anal verge was chosen as one variable for local recurrence risk factor analysis, because these low cancers are known to carry an increased risk for local recurrence [17].

Postoperatively, 31 patients received adjuvant therapy for 6 months and 4 patients chemoradiation. The mainly used adjuvants were single capecitabine and oxaliplatin with capecitabine, alone or with bevacizumab.

The rectal cancer patients were followed up according to a predetermined schedule. The healing of colorectal anastomosis was evaluated after 6 weeks by fiberosigmoidoscopy. After this, the follow-up visits were held biannually for the first 2 years and annually thereafter for up to 5 years. Fiberosigmoidoscopy or colonoscopy was performed at every visit up to 2 years, and colonoscopy was performed at 5 years. The levels of hemoglobin and carcinoembryonic antigen (CEA) were measured at every follow-up visit. A CT or MRI was taken only when disease recurrence was suspected based on clinical examination or symptoms, because the radiological examinations were not included in the routine follow-up protocol during the study period.

### Statistical analysis

The cumulative survival and the cumulative local recurrence risk were calculated by the Kaplan-Meier method. The potential risk factors for local recurrence were analyzed by the univariate Cox regression test. In multivariate analysis, tumor distance, mucinous histology, and lateral margin were included into the model, based on our previous article [17]. In addition, preoperative short-course RT was included. Differences between the two patient groups were analyzed by using Fisher’s exact test (dichotomous), the Mann-Whitney- test (continuous), and linear-by-linear test (ordinal by ordinal). Exact 95% confidence intervals (CIs) were calculated for difference in proportions. *p* values below 0.05 were considered significant, and all statistical tests were two-sided. Statistical analyses were run on SPSS version 23.0 (SPSS, IBM, Armonk, NY, USA) and StatExact version 4.0 (Cytel Software corporation, Cambridge, MA, USA).

## Results

### Median follow-up and survival rates

The median follow-up time was 4.3 (range 0.01–11.3) years for the whole patient population, 4.0 (0.2–11.2) years for the surgery only group and 4.4 (0.01–11.3) years for the RT plus surgery group. Fifty-one patients (33.8%) died during the follow-up period, 37 (24.5%) for cancer-related reasons. The 30-day postoperative mortality was 0.7%.

The cumulative overall survival at 1-, 3-, and 5-year in the whole 151 patient population was 92.7%, 81.1%, and 68.9%. In separate groups, the overall 1-, 3-, and 5-year survivals were 94.7%, 78.6%, and 59.2% for the surgery only group and 91.5%, 82.6%, and 75.2% for the RT plus surgery group (*p* = 0.13). The cancer-related overall 1-, 3-, and 5-year survival in the whole 151 patient population was 94.6%, 86.9%, and 73.9%. In separate groups, the cancer-related 1-, 3-, and 5-year survival was 98.2%, 87.1%, and 65.6% for the surgery only group and 92.5%, 86.7%, and 79.0% for the RT plus surgery group (*p* = 0.24).

### Local recurrence

**Fig. 1:**
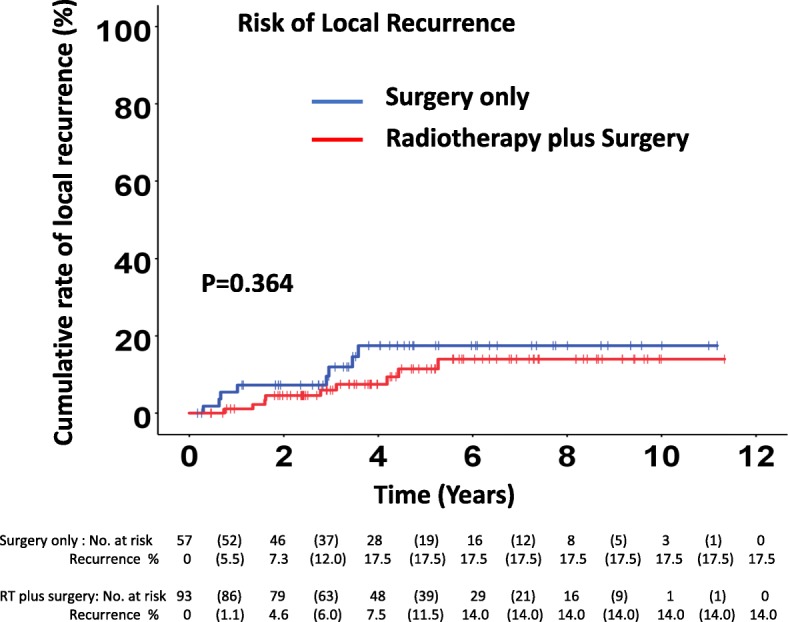
Cumulative risk of local recurrence between the surgery only and radiotherapy plus surgery groups in Kaplan-Meier analysis

Local recurrence afflicted 17 (11.3%) of 150 patients, 8 of 57 (14.0%) patients in the surgery only group and 9 of 93 patients (9.7%) in the RT plus surgery group (*p* = 0.44). The difference of proportions between the groups is − 4.3% (95% CI − 21.9 to + 8.6%). The median time for local recurrence in 17 patients was 2.8 (0.3–5.3) years. The median time to local recurrence was 2.0 (range 0.3–3.6) years for the surgery only group and 2.8 (0.7–5.3) years for the RT plus surgery group (*p* = 0.24). There was no significant difference in local recurrence between the surgery only and in RT plus surgery groups (*p* = 0.36) in the Kaplan-Meier analysis. The 2-, 3-, and 6-year cumulative risk for local recurrence was 7.3% (2 years), 12.0% (3 years), and 17.5% (6 years) in the surgery only group and 4.6% (2 years), 6.0 % (3 years), and 14.0% (6 years) for the RT plus surgery group (Fig. 1).

### Risk factor for local recurrence

Neoadjuvant short-course RT did not affect the risk of local recurrence after surgery of pT3N1-2M0 rectal cancer as analyzed in the univariate Cox regression model. The risk factors for local recurrence for the entire 150 patient population were the following: involved lateral margin (< 1 mm) in specimen (*p* < 0.001), tumor perforation (*p* < 0.001), tumor location under 6 cm from anal verge (*p* = 0.01), and mucinous histology (*p* = 0.006). Patient-related factors (gender, age, BMI), type of operation, 30-day anastomotic complications, invasion of vessels, nerves or lymphatics, or preoperative short-course radiotherapy did not affect the risk of local recurrence (Table 3). The risk factors for local recurrence analyzed in the multivariate analysis were the following: involved lateral margin (*p* < 0.001) and tumor location under 6 cm from anal verge (*p* = 0.03), but not neoadjuvant RT or mucinous histology. Also, subgroup analysis between males and females having tumor under 6 cm from the anal verge was calculated by Fisher’s exact test, and no significant difference between genders was noticed (*p* = 1.00).

**Table 3:** 95% confidence intervals Cox regression univariate and multivariate analysis of risk factors expressed as hazard ratios for local recurrence in 150 patients

Variable	Cox regression univariate analysis			Cox regression multivariate analysis		
	HR	95% Cl	*p*	HR	95% Cl	*p*
Gender (male)	1.14	0.44–3.00	0.79			
Age (≤ 65 years)	1.23	0.47–3.23	0.68			
BMI (≤ 25 kg/m^2^)	0.56	0.19–1.64	0.29			
Tumor ≤ 6 cm distance from anal verge	3.52	1.30–9.56	0.01	3.33	1.11–9.98	0.03
Preoperative short-course radiotherapy	0.65	0.25–1.67	0.37	0.58	0.22-1.55	0.28
APE compared to AR operation	1.19	0.34–4.16	0.78			
Mucinous histology	3.99	1.47–10.80	0.006	0.60	0.60–5.92	0.28
Involved lateral margin	7.90	2.75–22.74	< 0.001	6.40	2.01–20.36	0.002
Tumor perforation	8.31	2.70–25.53	< 0.001			
Invasion of vessels/nerves/lymphatics	2.43	0.93–6.33	0.07			
Early anastomotic complication	2.44	0.70–8.49	0.16			
Grade 1–2/3–4	1.57	0.45–5.53	0.48			

*HR* hazard ratio, *CI* 95% confidence interval, *BMI* body mass index, *AR* anterior resection (included Hartmann’s procedure), *APE* abdominoperineal excision

## Discussion

We found that neoadjuvant short-course RT was not a preventive factor for local recurrence in patients operated for pT3N1-2M0 rectal cancer in our dataset. Anyhow, all surgical procedures executed in the present study used the TME-technique, which is fundamental for the adequate dissection in a rectal cancer operation [7], and has better local control compared to blunt dissection [18]. Our data comprised only of patients with stage III diseases assessed by pathology report postoperatively. The total local recurrence rate for all the patients was 11.3%, for the RT plus surgery group 9.6% and for the surgery only group 14.0%. Thus, these findings are in line with a previously published local recurrence rate [19] and the difference between groups was not significant. A Dutch trial found that the local recurrence rate at 5 years was 9.3% for the RT plus surgery group and 19.4% for the surgery only group in stage III disease [20]. A Norwegian study, reporting the results of T3 rectal cancers treated with curative intention with TME without neoadjuvant treatment, observed a 3-year local recurrence rate to be about 18% for T3N1 tumors and about 24% for T3N2 tumors [9]. Possibly, the quality of TME in the early phase of that study, i.e., when TME was novel, in some of the centers might not have been at as high level as would be the case nowadays. Moreover, a series that included patients operated on before and after the adoption of the TME technique found the local recurrence risk for T3N1-2 patients to range between 34.5 and 42.9% [21].

The median time to local recurrence in our series seemed to be shorter in the surgery only group than for the RT plus surgery group (2.0 vs. 2.8 years). A similar pattern was also reported in the Dutch TME trial [20]. However, more patients in the RT plus surgery group in our study received adjuvant chemotherapy than in the surgery only group (87% vs. 61%), which may also have delayed the emergence of local recurrence. In our unit, patients with evidence of positive lymph node involvement in their specimen are evaluated by an oncologist for possible suitable adjuvant treatment, but not everyone receives chemotherapy because of patient-related factors, such as fragileness or prolonged recovery or refusal of such treatment by some patients.

Several studies have tried to separate good prognosis T3 tumors from poor prognosis T3 disease, both before and after TME era [8, 9, 21]. Those T3 tumors, predicted to have less than 5 mm spread from the muscularis propria in MRI, have a minor risk for local recurrence after surgery without RT, regardless of lymph node involvement status [8, 21]. In the early days of this study period, the T3 tumors were not divided into four subclasses by an MRI radiologist; thus, we do not know the distribution to rT3a-d subclasses. However, there were more tumor perforations in the non-irradiated group, which suggests that tumors in this group were locally more advanced.

In our study, 38% of pT3N1-2 patients did not receive neoadjuvant RT, even though our treatment strategy has been to irradiate T3 tumors when there is suspicion of lymph node involvement upon MRI. There are several reasons for this. First, the MRI diagnosis of lymph node involvement is difficult. Only half of the “good” prognosis patients with positive lymph node involvement in the MERCURY study had been evaluated to have had positive lymph node involvement in the preoperative MRI [8]. Second, some of our patients had received previous pelvic irradiation for other reasons and the neoadjuvant RT for rectal cancer was thereby contraindicated. Third, some other patient-related factors, such as difficult dementia or otherwise poor cooperation, may have been a reason not to offer RT.

The main risk factors for local recurrence in this specific group, pT3N1-2M0 rectal cancer in our analysis, were as follows: involved CRM, tumor distance under 6 cm from anal verge, and tumor perforation. All these risk factors for local recurrence have already been acknowledged in many studies [3, 9, 22] and also in the data of our earlier published study [17]. A Dutch study reported the risk factors for local recurrence to be stage IV disease, T4 tumor, positive CRM in T3 or T4 tumors, and also N2 disease [20]. The preoperative RT did not prevent local recurrence in our study patients with stage III disease.

One reason for the critical evaluation of the usefulness of preoperative RT in this setting is its possible toxicity. Low anterior resection syndrome and poor functional outcome occur more often in patients who receive RT compared to patients whom have had surgery only [10, 13]. A recovery to daily activity is also slower after having received RT. Sexual dysfunction is a problem, as well [11, 12, 23]. Radiotherapy is found to be a risk factor for anastomotic leakage, which in itself may prevent adjuvant chemotherapy and also adversely affect bowel function [14]. The wound healing after abdominoperineal excision (APE) is slower after RT than without it [3, 24]. More secondary malignancies are reported to occur after RT (9.5% compared surgery only 4.3%) in irradiated areas [15]. More than 50% of patients report long-term side effects after having RT [23].

In the light of the data available including those of the present study, the efficacy of neoadjuvant short-course RT in T3N+ rectal cancer is uncertain and needs to be studied more. Radiotherapy decreased the amount of local recurrences before the TME era [1]. During the learning phases of TME surgery soon after its adoption, RT still had a beneficial effect on the local recurrence rate of tumors with positive lymph node involvement, at least in multicenter studies [3, 4]. It is also possible that multicenter studies [3, 4] also included centers of lower technical quality. However, other more current studies showed that even lower recurrence rates can be achieved by using meticulous TME surgery alone and the results were not poorer for lymph node positive disease, at least in good prognosis T3 tumors [7, 8]. The adoption of neoadjuvant short-course RT in rectal cancer treatment has taken place within the same time period as TME technique, and MRI-directed multidisciplinary teams in designing the treatment for each patient have become a standard, both of which have been shown to improve oncological results [2, 18, 22, 25]. T3c-d tumors seem to have poorer prognosis in terms of local recurrence, but it is still not known whether that subgroup of tumors would eventually benefit from preoperative short-course RT.

Our current data on pT3N1-2 tumor outcome suggests that the routine use of neoadjuvant short-course RT in T3N+ rectal cancer patients may not be justifiable for all patients. However, patient number in our study was small and patient groups may not be quite equivalent due to retrospective nature of the study. Thus, the possible beneficial effect of neoadjuvant RT may not manifest. Further, due to limited number of patients in our study, different T3 subgroups (T3a–c) as well as N1 and N2 patients could not be separately studied. Thus, some subgroup possibly having advantage of short-course radiotherapy could not be recognized.

In conclusion, neoadjuvant short-course RT did not beneficially affect the local recurrence rate of pT3N1-2M0 rectal cancer in our retrospective series. Since short-course RT also has potential side effects, its role as routine neoadjuvant treatment in T3N+ disease should be critically considered. However, further studies with larger patient numbers on pT3b-c rectal cancer and in N1 and N2 disease are needed to evaluate the value of neoadjuvant therapy in these particular groups.

## Data Availability

The datasets used and/or analyzed during the current study are available from the corresponding author on reasonable request.
